# Kinetic and Kinematic Features of Pedestrian Avoidance Behavior in Motor Vehicle Conflicts

**DOI:** 10.3389/fbioe.2021.783003

**Published:** 2021-11-25

**Authors:** Quan Li, Shi Shang, Xizhe Pei, Qingfan Wang, Qing Zhou, Bingbing Nie

**Affiliations:** State Key Lab of Automotive Safety and Energy, School of Vehicle and Mobility, Tsinghua University, Beijing, China

**Keywords:** pedestrian safety, active behavior, kinematics, biomechanics, integrated safety, volunteer testing

## Abstract

The active behaviors of pedestrians, such as avoidance motions, affect the resultant injury risk in vehicle–pedestrian collisions. However, the biomechanical features of these behaviors remain unquantified, leading to a gap in the development of biofidelic research tools and tailored protection for pedestrians in real-world traffic scenarios. In this study, we prompted subjects (“pedestrians”) to exhibit natural avoidance behaviors in well-controlled near-real traffic conflict scenarios using a previously developed virtual reality (VR)-based experimental platform. We quantified the pedestrian–vehicle interaction processes in the pre-crash phase and extracted the pedestrian postures immediately before collision with the vehicle; these were termed the “pre-crash postures.” We recorded the kinetic and kinematic features of the pedestrian avoidance responses—including the relative locations of the vehicle and pedestrian, pedestrian movement velocity and acceleration, pedestrian posture parameters (joint positions and angles), and pedestrian muscle activation levels—using a motion capture system and physiological signal system. The velocities in the avoidance behaviors were significantly different from those in a normal gait (*p* < 0.01). Based on the extracted natural reaction features of the pedestrians, this study provides data to support the analysis of pedestrian injury risk, development of biofidelic human body models (HBM), and design of advanced on-vehicle active safety systems.

## 1 Introduction

Road traffic injuries remain a major global health issue, resulting in the loss of 310,000 pedestrian lives each year and representing 23% of all road traffic deaths (WHO, 2018). Pedestrian pre-impact posture significantly influences the risks and severity of injury outcomes (Li et al., 2015; Zou et al., 2020). Understanding the active avoidance behavior of pedestrians in dangerous traffic scenarios is necessary to develop advanced pedestrian safety systems that can reduce the risk of injury. The traditional survey method used to investigate accidents involves collecting detailed information (such as the vehicle trajectory and injury distribution) after a collision and then reconstructing the scenario. However, it is not realistic to collect accurate information on pedestrian avoidance behavior using this method (Fredriksson et al., 2010). By watching and analyzing real-world accident video records, researchers have recently observed the emergency postures and kinematics of pedestrians in dangerous impact scenarios (Zou et al., 2020; Wu et al., 2021). However, the pedestrian avoidance behavior has not been quantified.

Pedestrian avoidance behavior affects the impact posture, which then affects the subsequent impact condition and injury risk variables (Simms and Wood, 2006; Tang et al., 2016). In previous studies, common methods for investigating pedestrian injuries have included traffic accident database analyses (Shang et al., 2018), cadaver impact tests (Shang et al., 2020), human surrogate crash tests (Konosu et al., 2005), and mathematical analyses based on numerical pedestrian models (Forman et al., 2015; Nie and Zhou, 2016). Understanding the active behavior of pedestrians during traffic conflicts is vital for predicting pedestrian injury risk under complex impact conditions. However, several previous studies have focused on analyzing pedestrian injuries under stationary or normal walking postures, while ignoring the influence of active pedestrian behaviors (EEVC, 1994); therefore, the conventional computational methods (e.g., multi-body (MB)/finite element (FE) modeling) in the field of vehicle safety does not fully represent the actual collision conditions. Some recent studies have investigated the influence of pedestrian avoidance behavior on injury risk through volunteer tests and simulations (Holdgrin et al., 2018). However, the biomechanical features of pedestrians, such as the kinetics and kinematics, during active avoidance remain unidentified.

Pedestrian will perform activation behavior such as acceleration forward or deceleration backward to avoid danger when they notice the upcoming vehicle in traffic conflicts. If the pedestrian does not notice the danger, they will keep walking with a constant velocity. Thus, the avoidance behavior of pedestrians affects the subsequent trajectories, which shall be considered by the on-vehicle active safety system. Previous studies have focused on predicting normal pedestrian walking trajectories in safe traffic scenarios owing to the limited sample size for dangerous-scenario datasets (Rasouli et al., 2018). The randomness of pedestrian avoidance behavior remains a major challenge for vehicle awareness systems in real-world accidents (Zhang et al., 2019). Therefore, analyzing and quantifying pedestrian avoidance behaviors in dangerous scenarios is an important research topic in the context of advanced vehicle safety systems (Sportillo et al., 2018). Accordingly, the aims of this study are to 1) identify and quantify the kinetic and kinematic features of pedestrian active behavior in dangerous impact scenarios, 2) analyze and quantify pedestrian avoidance reactions to dangerous scenarios, and 3) quantify the pedestrian–vehicle interaction in the pre-crash phase.

## 2 Materials and Methods

### 2.1 Experimental Platform and Design

We developed a pedestrian natural response experimental platform and recruited volunteers to determine the kinetic and kinematic features of the subjects’ (“pedestrians”) active avoidance behavior. The experimental platform comprised three modules: a virtual reality (VR) test platform, a kinetic capture system, and an electromyogram (EMG) signal capture system. Specifically, the VR test platform (51VR High Technology Co., Ltd.) generated a near-real immersive VR traffic scene. The peripheral auditory functions, sense of distance, and interactions of the subjects immersed in the virtual traffic environment functioned as normal. The subjects performed the action of crossing the road in the VR traffic scene, while a dangerous scenario was created to produce a natural reaction in the subjects. For representing typical conflict scenes between pedestrians and vehicles in real-world (Han et al., 2017) and stimulating pedestrians to respond naturally, one of two emergent near-real car–pedestrian conflict scenarios (traffic scene A (TSA) and traffic scene B (TSB)) was created by the experimenter immediately after the subject entered the road. Detailed information on the experimental platform can be found in our previous studies (Li et al., 2020; Nie et al., 2021). To record the natural responses of the pedestrians and eliminate the influence of vigilance on crossing behavior caused by repeated experiments, each subject participated only once per scene. An experiment video is accessible through the following link: https://github.com/QuanLI-21/Pedestrian-avoidance-behavior-dataset-PABD.

### 2.2 Subjects and Ethical Statement

The experimental procedures were approved by the Institutional Review Board (IRB) of Tsinghua University. The procedures were performed according to the approved guidelines. Informed consent was obtained from each subject before conducting the experiments. A total of n = 24 subjects were recruited, conforming to the inclusion criteria for this study. However, due to the calibration error of the motion capture system and the wireless connection failure of the VR glasses, only n = 34 experiments (involving 19 subjects) were recorded completely and used in the final data analysis. The human body information for the subjects is provided in Supplementary Table S1 **.**

### 2.3 Feature Extraction of the Pedestrian Avoidance Behavior

#### 2.3.1 Coordinates of the Pedestrian and Vehicle

**FIGURE 1:**
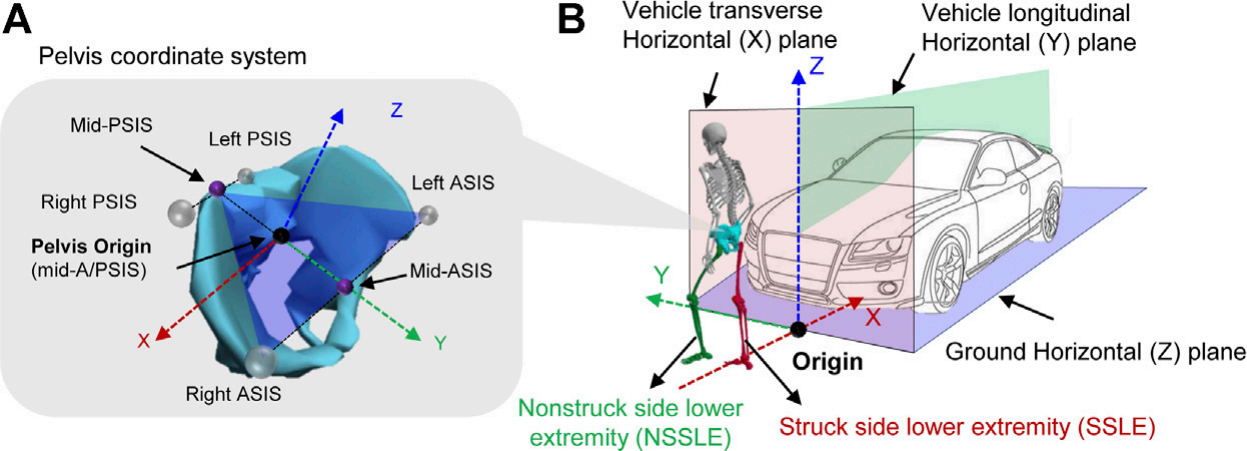
Illustration of the locations of the coordinate system; **(A)** pedestrian local coordinate system, **(B)** vehicle coordinate system.

The dynamic relative positions of the pedestrian and vehicle during the interaction were extracted using a motion capture system. The pedestrian moved in the vehicle coordinate system, and the pedestrian’s local coordinate system was located on the pelvis (Figure 1).

#### 2.3.2 Pedestrian Kinetics and Kinematics

The pedestrian kinetic and kinematic features include the relative locations of the vehicle and pedestrian, the pedestrian’s movement velocity and acceleration, their posture (joint positions and angles), and their muscle activation levels. The data were recorded and extracted using the 12 cameras (100 Hz) of the motion capture system (No. Mars 2H; Beijing Nokov Science and Technology Co., Ltd.) by tracking 54 markers. In addition, 11 joints were used to describe the pedestrian posture (Arnold et al., 2010) (Supplementary Figure S1). The pedestrian kinematics were inversed using OpenSim and the “Full Body Model” (Rajagopal et al., 2016).

The EMG signals of the lower limb surface muscles were recorded using a physiological signal system (16 channels, DELSYS Trigno wireless system). The EMG signals of the lower extremities were measured using eight separate electrical signal sensors (sampling frequency: 2000 Hz). Considering the relationship between the lower limb muscles and motion, the rectus femoris (RF), vastus lateralis (VL), vastus medialis (VM), biceps femoris (BF), gastrocnemius medial head (GMH), gastrocnemius lateral head (GLH), soleus (SO), and tibialis anterior (TA) muscles were selected for testing (Supplementary Figure S2). The raw EMG signals were band-pass-filtered (20–500 Hz), full-wave-rectified, and smoothed with a 25 ms root mean square window. The muscle activation levels were calculated based on the normalized EMG signals through a parallel experimental process based on maximum voluntary contraction (MVC) signals. The experimental approach for the MVC calculation is illustrated in Supplementary Figure S3.

In addition, data analysis was performed using MATLAB 2019b. The Kruskal–Wallis test was used to detect differences in pedestrian avoidance behaviors.

## 3 Results

We categorized four representative pedestrian behaviors based on the direction of the avoidance motion. The avoidance processes of pedestrians in vehicle–pedestrian conflicts are illustrated in detail through case studies. We fitted the average velocities, pre-crash postures, acceleration of pedestrians, and relative locations of the vehicle and pedestrian. These results can assist in understanding and quantifying the kinetics and kinematics of pedestrian active avoidance behaviors. The experimental data, such as the EMG, kinematics, and video recordings, are available online at Github repositories (link in section 2.1).

### 3.1 Representative Reaction Categories in Vehicle–Pedestrian Conflicts

The test subjects exhibited representative avoidance behaviors to avoid the “bullet vehicle” in car–pedestrian conflicts. The “bullet vehicle” refers to a vehicle which would suddenly appear and virtually crash into the pedestrian. The results of the conflicts and pedestrian reactions in all cases are summarized in Supplementary Table S2. In our previous study, the actions were classified into four categories based on the perception of the “bullet vehicle” and the relative motion vector: (1) backward avoidance (BA), (2) forward avoidance (FA), (3) oblique stepping (OS; startled response), and (4) walking/no avoidance reaction (NAR; not noticing the approaching vehicle). Based on the avoidance behavior categories, we discuss the kinetic and kinematic features of pedestrian avoidance behaviors in detail through case studies.

### 3.2 Case Studies

To explain pedestrian kinetic and kinematic features in avoidance behavior, we randomly selected and analyzed one collision case from each of the three avoidance behavior categories. We considered the relative locations of the vehicle and pedestrian, posture, kinematics, and EMG signals in the process of the avoidance behavior. The three representative avoidance processes in the VR environment and in-lab environment are shown in Supplementary Figure S4. In addition, one case in which the pedestrian walked normally (NAR case, Supplementary Figure S5) was compared with the cases in the other categories (Figures 3–5) to illustrate the differences in the kinetics and kinematics.

#### 3.2.1 Backward Avoidance Case

**FIGURE 2:**
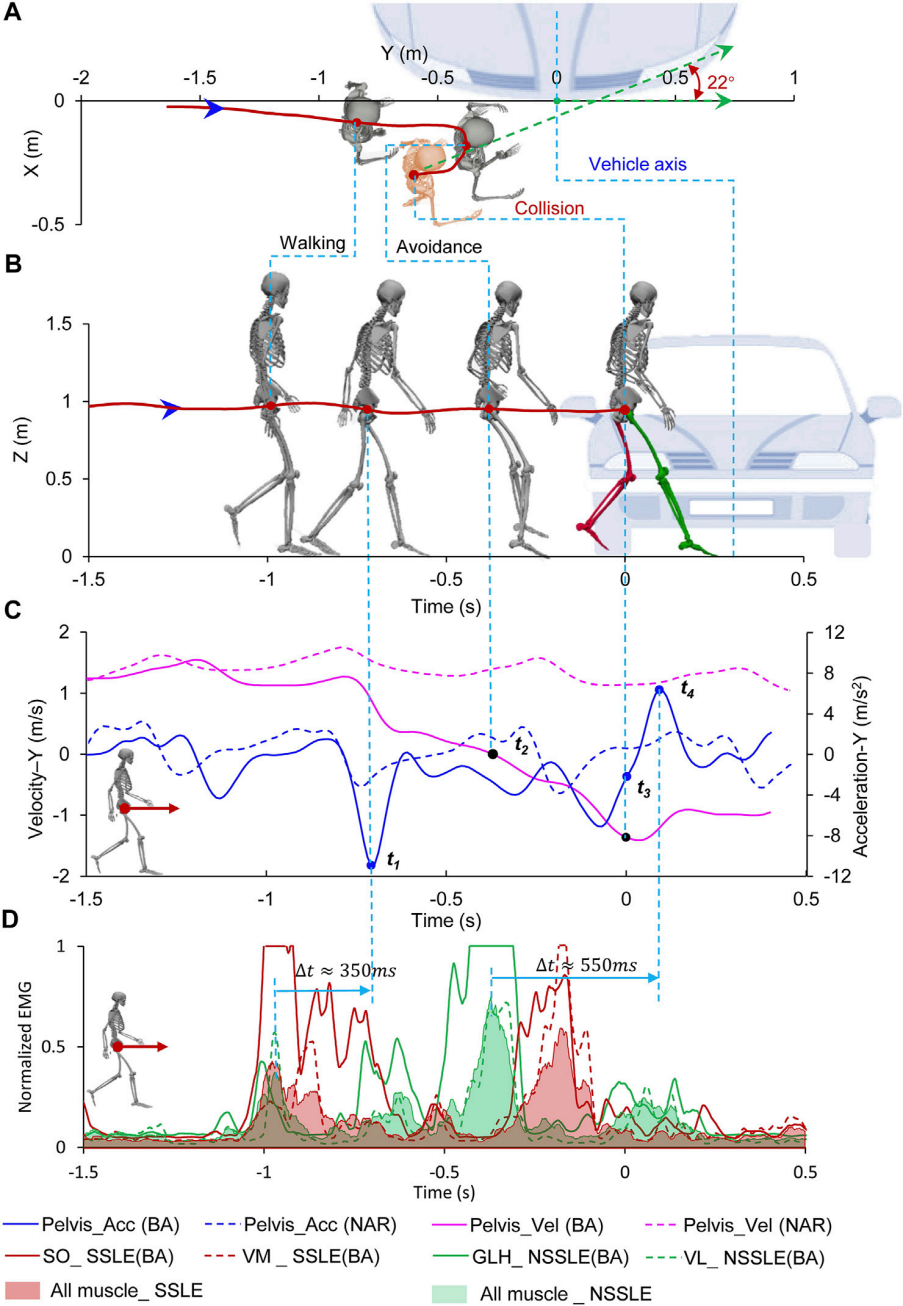
Pedestrian backward avoidance behavior. **(A)** Top view of relative location of pedestrian and vehicle; **(B)** Side view of relative location of pedestrian and vehicle; **(C)** Pedestrian’s acceleration and velocity during avoidance, the dotted line represents the process of the pedestrian walking normally (Supplementary Figure S5); **(D)** Muscle activation level of pedestrian during avoidance. Time = 0 s represents the collision time. The location of the vehicle at the collision time is shown. Negative times represent the time before the virtual collision.

In this case (subj019_TSB), the subject entered the vehicle lane before the collision and started to step back to avoid the impending collision upon noticing the “bullet vehicle”; the collision occurred in the process of the subject stepping backward (Figure 2; Supplementary Figure S4A). According to the analysis of the trajectory of the subject (Figures 2A,B), the subject exhibited an avoidance behavior of stopping and moving backward. The velocity, acceleration, and myoelectric signals of normal pedestrian walking are presented in Figures 2C,D. Eventually, the subject rotated 22° toward the vehicle (Y-direction of the pelvis and vehicle coordinate system) and collided with the vehicle at y = -0.6 m (left side of the vehicle) from the vehicle’s center axis (Table 1). In this avoidance process, the subject initially decelerated forward and then accelerated backward; and the peak values of the pelvis deceleration and acceleration in the Y-direction were −10.9 and 6.4 m/ s^2^, respectively, which is significantly higher than the peak acceleration observed during the normal walking test (Figure 2C). Time histories (*t*_*1*_ *, t*_*2*_ *, t*_*3*_ *, t*_*4*_) of the posture information for subjects are provided in Supplementary Table S3 **.**

**TABLE 1:** Pelvis kinematics at the instant of collision in the case studies.

Pelvis kinematics	Backward avoidance	Forward avoidance	Oblique steeping avoidance
$\mathrm{Ve}l_{x}\;\mathrm{(m}/s)$	-0.1	-0.8	-1.5
$\mathrm{ve}l_{y}\cdot \mathrm{(m}/s)$	-1.4	2.8	0.9
$\mathrm{Ve}l_{z}\;\left(m/s\right)$	0.2	0.3	0.1
$\mathrm{Ac}c_{x}\;\left(m/s^{2}\right)$	-1.2	-4.3	-0.9
$\mathrm{Ac}c_{y}\;\left(m/s^{2}\right)$	-2.1	-2.1	6.4
$\mathrm{Ac}c_{z\;}\left(m/s^{2}\right)$	-5.9	-10.2	4.2

$Vel_{x}$ , $Vel_{y}$ , $Vel_{z}$ , $Acc_{x}$ , $Acc_{y}$ , and $Acc_{z\;}$ represent the velocity and acceleration of the pelvis in the X-, Y-, and Z-directions, respectively.

With regard to the kinetics, the lower limb muscles contract and control the foot striking the ground to gain momentum and drive the pelvis motion. Thus, myoelectric signals can represent muscle activation and capture the pelvis motion features. The muscle activation time histories of the four most activated muscles are shown in Figure 2D. In the process of BA behavior, the SO and VM of the struck-side lower extremity (SSLE) as well as the GLH and LV of the non-struck-side lower extremity (NSSLE) showed higher activation states. Physiologically, the generation of movement was delayed compared with that of the muscle force, which, in turn, was delayed compared with that of the myoelectric signals. The delay time is affected by several factors, such as individual differences and movement characteristics (De Luca, 1997). Therefore, the delay times between the myoelectric signals and corresponding movement show significant discrepancies between individuals and motions. In this study, to facilitate the determination of lower limb muscle activation, the myoelectric signals measured from the SSLE and NSSLE were summed and normalized, respectively, and were significantly higher than the myoelectric signals recorded during normal walking; this indicate that the scenarios were effective. The delay times between the peak value of the myoelectric signals and the peak pelvis acceleration were approximately 350 and 550 ms in the processes of deceleration and acceleration, respectively.

The time interval also varies with individual differences and response behaviors. As it is difficult to determine the accurate time interval by single muscle or muscle group, the presented time intervals serve as a reminder and demo. It reminds that it is necessary to consider proper time intervals when using the EMG signal to characterize the muscle activation state and kinematics at a certain moment.

#### 3.2.2 Forward Avoidance Case

**FIGURE 3:**
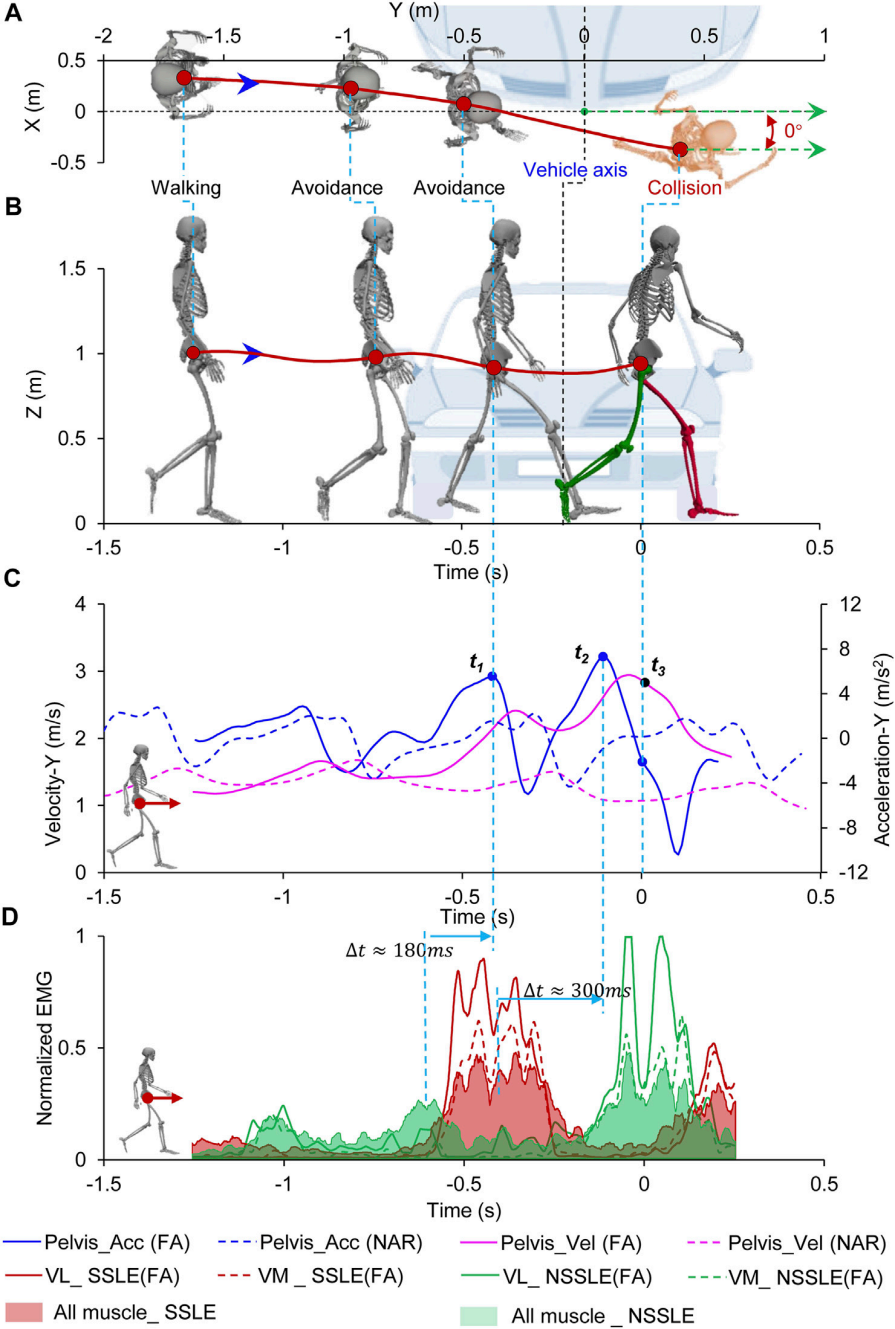
Pedestrian forward avoidance behavior process. **(A)** Top view of relative location of pedestrian and vehicle; **(B)** Side view of relative location of pedestrian and vehicle; **(C)** Pedestrian’s acceleration and velocity during avoidance, the dotted line represents the process of the pedestrian walking normally (Supplementary Figure S5); **(D)** Muscle activation level of pedestrian during avoidance. Time = 0 s represents the collision time. The location of the vehicle at the collision time is shown. Negative times represent the time before the collision.

In this case (subj014_TSB), the subject entered the vehicle lane before the collision and chose to avoid collision by accelerating across the road upon noticing the “bullet vehicle”; the collision occurred in the process of acceleration (Figure 3; Supplementary Figure S4B; Supplementary Table S4). According to the motion trajectory of the subject (Figures 3A,B), the subject performed a forward avoidance motion exhibiting a trend of moving away from the vehicle in the direction of vehicle movement. Eventually, in a running posture, the subject collided with the vehicle at y = 0.4 m (right side of the vehicle) from the vehicle’s center axis (Figure 3B; Table 1). In the process of forward avoidance, the subject underwent two accelerations through the NSSLE and SSLE striking the ground to gain a driving force, and the peak values of the pelvis acceleration in the Y-direction were 5.5 and 7.3 m/ s^2^, respectively (Figure 3C). In terms of the muscle activation state, the VL and VM of the SSLE and NSSLE showed higher activation levels, and the muscle activation level of the thighs was higher than that of the shank (Figure 3D). The myoelectric signals reached their peaks approximately 180 and 330 ms earlier than the pelvis accelerations did during the two accelerations. The peak values of the myoelectric signals and pelvis acceleration and deceleration were significantly higher than those observed in the normal walking test.

#### 3.2.3 Oblique Stepping Avoidance Case

**FIGURE 4:**
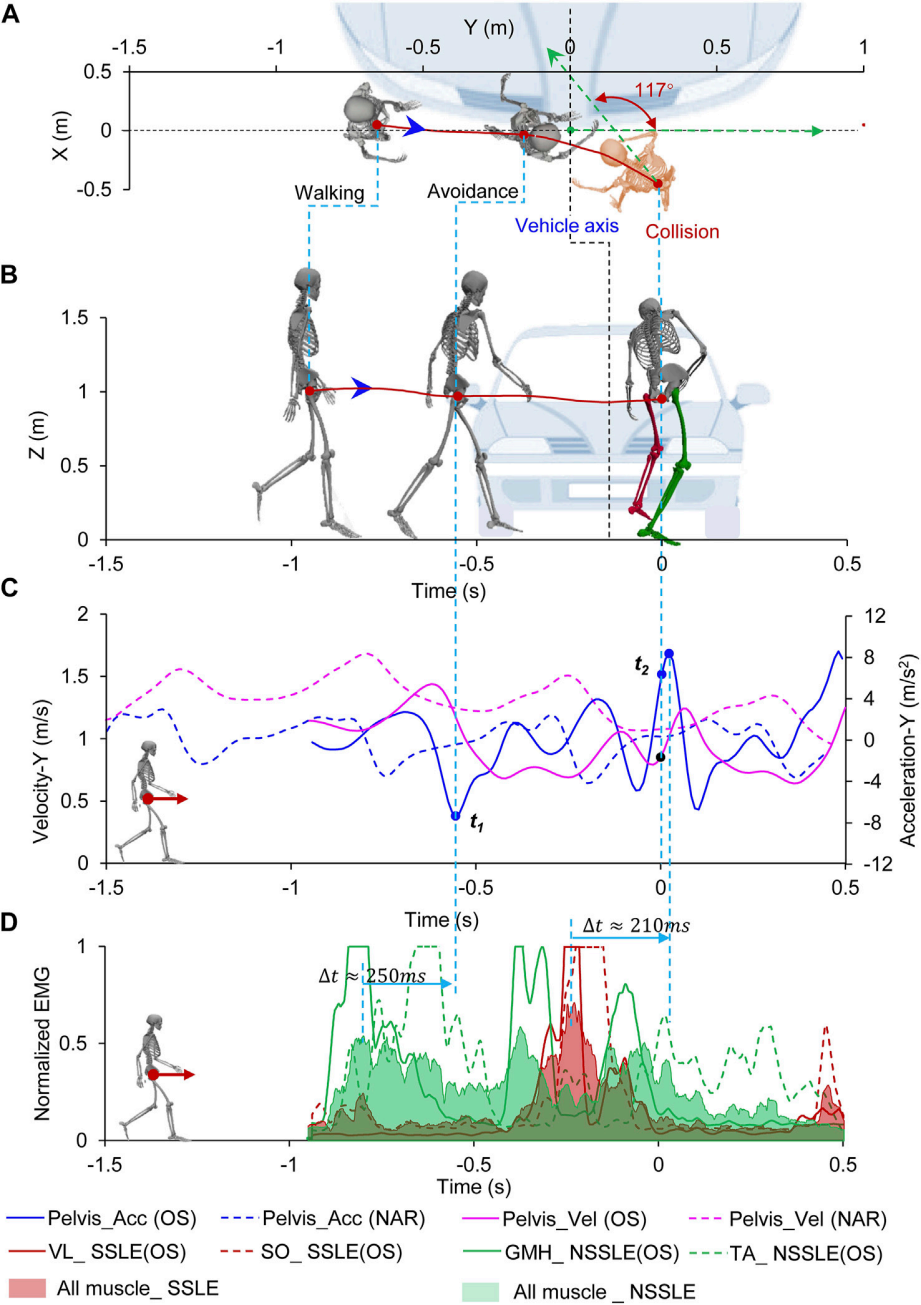
Pedestrian oblique stepping avoidance behavior process. **(A)** Top view of relative location of pedestrian and vehicle; **(B)** Side view of relative location of pedestrian and vehicle; **(C)** Pedestrian’s acceleration and velocity during avoidance, the dotted line represents the process of the pedestrian walking normally (Supplementary Figure S5); **(D)** Muscle activation level of pedestrian during avoidance. Time = 0 s represents the collision time. The location of the vehicle at the collision time is shown. Negative times represent the time before the collision.

In this case (subj016_TSA), the subject entered the vehicle lane before the collision and raised his hands in an attempt to stop the vehicle upon noticing the impending danger. The collision occurred in the process of oblique stepping avoidance (Figure 4; Supplementary Figure S4C; Supplementary Table S5). According to the trajectory of the subject’s motion (Figures 4A,B), the subject first underwent a forward deceleration and then turned to face the front of the vehicle while simultaneously moving away from the vehicle to avoid the collision. Eventually, while in a stepping posture rotated 117° toward the vehicle (Y-direction of the pelvis and vehicle coordinate system), the subject collided with the vehicle at y = 0.3 m (right side of the vehicle) from the vehicle’s central axis (Figure 4B; Table 1). In the OS avoidance process, the subject underwent a deceleration because of the SSLE striking the ground first, followed by the NSSLE striking the ground to create an oblique acceleration; the peak values of the pelvis deceleration and acceleration in the Y-direction were −7.3 and 8.4 m/ s^2^, respectively (Figure 4C). The four muscles with the highest activation levels during the OS behavior were the LV and SO of the SSLE and the GMH and TA of the NSSLE (Figure 4D). The peak values of the myoelectric signals occurred approximately 210 and 250 ms after the peak value of the pelvis acceleration during the acceleration and deceleration, respectively. Similar to the previous case results, the peak values of the myoelectric signals, pelvis acceleration, and deceleration were significantly higher than those of normal walking.

In addition, we used the Kruskal–Wallis test to detect differences in the kinematic features between different avoidance behaviors and normal walking. The results showed that the velocities were significantly different between the avoidance behaviors and normal gait (*p* < 0.01 for BA, FA, and OS vs. NAR).

### 3.3 Kinetic and Kinematic Features of the Pedestrian

Vehicle–pedestrian collisions usually occur with a process of pedestrian avoidance, which results in a highly random pedestrian impact posture. The average kinetic and kinematic features of the pedestrian “pre-crash postures” corresponding to the peak values of the pedestrian pelvis acceleration during the motion were extracted. With regard to the kinematic features of the pedestrian “pre-crash posture,” the overall kinematics of the pedestrian’s body were represented by the pelvis motion and posture information, which included the time histories of the velocity and acceleration, joint angles, and joint coordinates. The kinetic features of the pedestrian avoidance behavior were extracted from the EMG signal of the lower limb muscles to represent the muscle activation and exertion.

#### 3.3.1 Average Kinematic Features and “Pre-crash Postures” of Pedestrians

**FIGURE 5:**
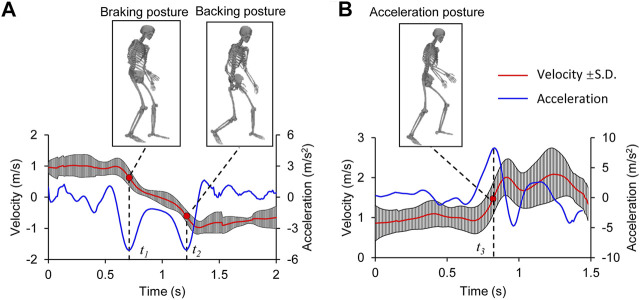
Pelvis velocity corridors and “pre-crash postures” for different motion categories; **(A)** backward avoidance, **(B)** forward avoidance; *t*_*1*_, *t*_*2*_, and *t*_*3*_ are the moments of peak acceleration in the processes of braking, backing, and acceleration, respectively. The peak acceleration occurrence was defined as a time alignment standard for the participants under the same motion category.

The BA behavior had a two-phase motion: deceleration to stop followed by backward acceleration (Figure 5A). The subjects required approximately 1.0 s to decelerate from the initial velocity of approximately 1 m/ s to the backward velocity of 1 m/ s. The braking and backing postures at the occurrence of the peak accelerations during deceleration ( $t_{1}$ ) and acceleration ( $t_{2}$ ) were extracted as the “pre-crash postures.” The FA behavior included a consistent forward acceleration motion from the initial velocity of approximately 1 m/ s to a forward velocity of 2 m/ s (Figure 5B). The acceleration posture at the occurrence of the peak acceleration during acceleration ( $t_{3}$ ) was extracted. The “pre-crash posture” was normalized by the cases with the same motion categories and illustrated with the joint angles of the human body (Eq. 1); the joint angles of the three “pre-crash postures” are listed in Supplementary Table S6.

$$AJ_{i}=\frac{1}{n}\sum_{j=1}^{n}J_{ij},$$ (1)

where $AJ_{i}$ denotes the average angle of subject joint i , $J_{ij}$ denotes the angle of subject joint *i* in case *j*, and *n* denotes the number of cases.

**FIGURE 6:**
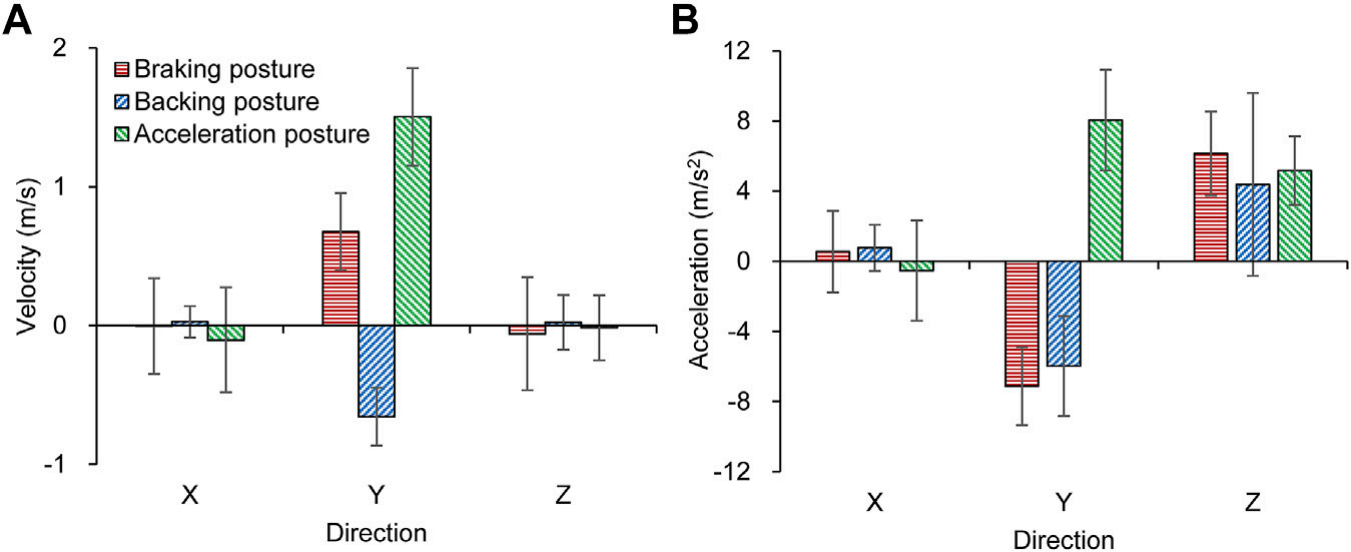
Peak values of the kinematic indicators for the “pre-crash postures”; **(A)** Pedestrian pelvis velocity, **(B)** Pedestrian pelvis acceleration.

Corresponding to the three aforementioned “pre-crash postures,” the average peak velocities in the Y-direction for the braking posture, backing posture, and acceleration posture were −0.7 m/ s, 0.7 m/ s, and 1.5 m/ s, respectively (Figure 6A). The velocities of the pedestrian in the X-direction and Z-direction were approximately 0 m/ s. The average peak accelerations of the braking posture, backing posture, and acceleration posture in the Y-direction were −6.0 m/ s^2^, −7.1 m/ s^2^, and 8.1 m/ s^2^, respectively (Figure 6B). In addition, the three “pre-crash postures” exhibited a positive acceleration in the Z-direction, with average peak values of 6.2, 4.4, and 5.2 m/ s^2^, respectively.

#### 3.3.2 Muscle Activation Levels for “Pre-crash Postures”

**FIGURE 7:**
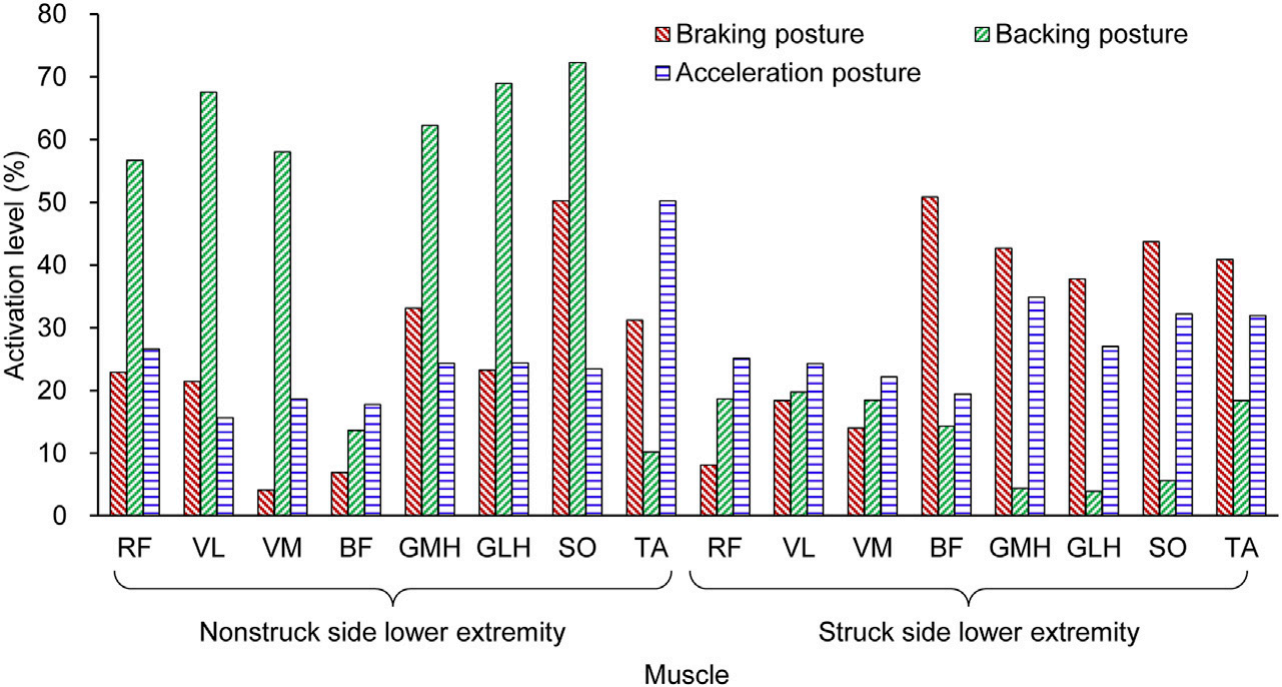
Muscle activation levels in pedestrian avoidance postures.

Different muscle groups were activated on the SSLE and NSSLE. For the three average pre-crash postures mentioned before, the muscle activation levels of the lower limbs were normalized based on the results of the MVC tests. (Soni et al., 2013b) (Figure 7). For the braking posture, the NSSLE struck the ground to gain momentum for braking, and the SSLE stopped swinging forward. The muscles in both the SSLE and NSSLE were activated to varying degrees. Similarly, for the backing posture, the NSSLE, as the supporting leg, struck the ground to gain momentum for backward avoidance, and the muscle activation level was higher than that of the SSLE. For the acceleration posture, the NSSLE struck the ground, and the SSLE swayed forward rapidly.

#### 3.3.3 Pedestrian Trajectory During Vehicle Interaction

**FIGURE 8:**
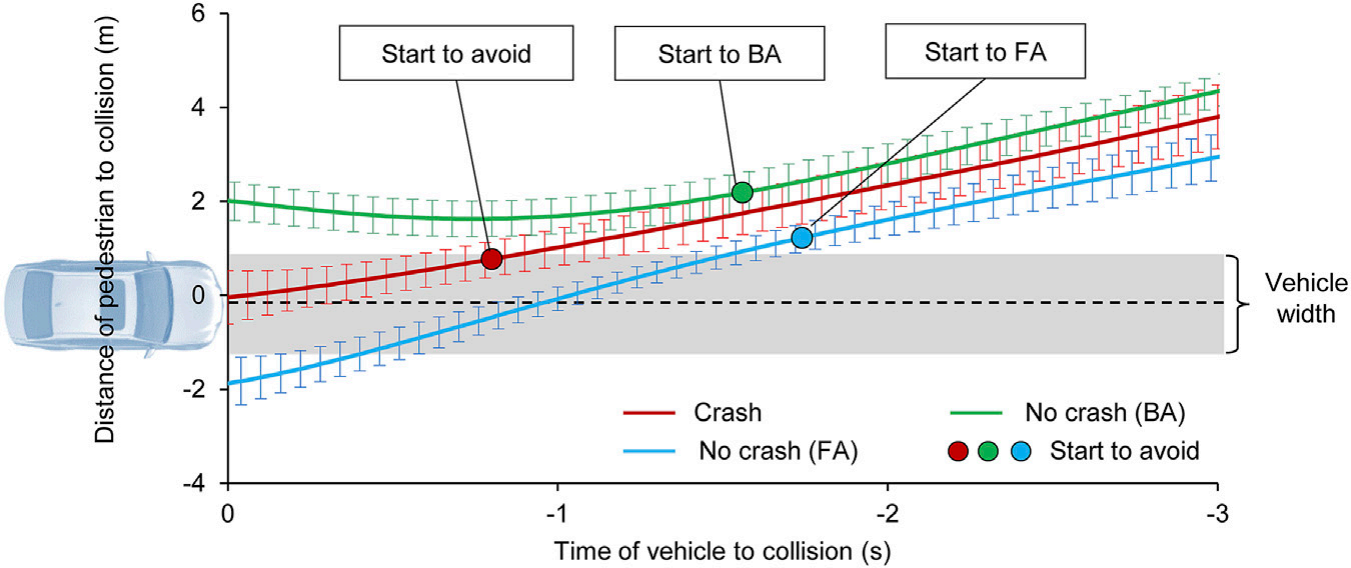
Relative locations of the vehicle and pedestrian during the interactions. The red curve represents the crash cases, in which the pedestrian noticed the coming vehicle but failed to avoid the virtual collision. The green curve indicates that the pedestrian successfully avoided the collision through backward avoidance. The blue curve indicates that the pedestrian successfully avoided the collision through forward avoidance. The standard deviation of the data is also shown.

The pedestrian trajectories relative to the “bullet vehicle” during the collisions are shown in Figure 8. The vertical axis represents the distance between the pedestrian and the vehicle lane, and the horizontal axis represents the time when the vehicle reached the potential collision location. According to the average values of the experiment results, when the pedestrian noticed the “bullet vehicle,” 1) if the subject was located 2.2 m (SD 0.25) away from the vehicle center lane and the time to collision (TTC) was longer than 1.6 s (SD 0.38), the collision could be avoided by stepping backward; 2) if the subject was located approximately 1.2 m (SD 0.97) away from the vehicle center lane and the vehicle TTC was more than 1.8 s (SD 0.64), the collision could be avoided by performing the forward avoidance motion. In the collision cases, the subjects noticed the “bullet vehicle” too late to avoid collision in a short time window.

## 4 Discussion

We investigated and quantified the kinetic and kinematic features of active pedestrian behaviors in virtual dangerous impact scenarios. The observed natural avoidance behaviors were similar to those in real-world accidents (Schachner et al., 2020). The results can thus represent natural pedestrian behaviors, help us better understand the pedestrian behavior features in real-world accidents, and facilitate development of an advanced integrated safety system that combines active and passive functions.

### 4.1 Kinetic and Kinematic Features of Pedestrian Avoidance Behavior

Pedestrians are individuals with active behavior abilities and awareness; their active avoidance behavior will be activated when they notice an approaching vehicle with a potential collision risk. Consequently, pedestrians will exhibit forward, backward, jumping, and other non-standing behaviors when facing danger. However, the existing warning systems for pedestrian safety only focus on vehicle avoidance behavior (Matsui et al., 2013) and default pedestrians to uniform movement or stationary positions; thus such systems fail to consider active pedestrian behaviors (Hamdane et al., 2015). To predict the collision risk as a follow-up study, two key points are addressed: 1) quantifying pedestrian avoidance abilities in a broad range of conditions, and 2) identifying the impact conditions of pedestrians and vehicles during inevitable collisions. This study provides a new viewpoint for evaluating collision risk from the perspective of pedestrians. The minimum reaction time of pedestrians who avoided collision was less than 1.6 s in BA behaviors and 1.8 s in FA behaviors when they noticed the approaching vehicle in advance (Figure 8). Active behavior usually relies on surrounding visual and auditory information (Koh et al., 2014). Therefore, when emphasizing the improvement of vehicle collision risk prediction and avoidance capabilities, measures to activate pedestrian avoidance capabilities, such as automatic whistles, are needed.

Predicting the trajectory of a pedestrian in a real-world environment is challenging because of the randomness of natural human reactions. The active behavior of pedestrians depends on whether they notice the vehicle, as well as the relative locations of the vehicle and pedestrian, and identifying this active behavior is vital for advanced pedestrian safety warning systems. Pedestrian active avoidance behaviors were characterized in this study and can provide a reference for further pedestrian behavior prediction.

### 4.2 Influence of Pedestrian Avoidance Behavior on Potential Injury Risk

We collected kinetic and kinematic data for natural pedestrian avoidance reactions in vehicle conflicts, and the vehicle–pedestrian interaction processes were analyzed in detail using case studies. A previous study identified several influencing factors for the injury risk of pedestrians with a normal gait in vehicle crashes, such as stature, impact posture, orientation, and obesity (Elliott et al., 2012; Tang et al., 2020b). However, the posture, kinetics, and kinematics of the pedestrians at the time of collision were significantly different from those of the normal gait (Supplementary Figure S4). Such difference would result in different injury risks between the pedestrians with active avoidance posture and normal gait if the collision occurs. In the BA case (Figure 2B; Table 1), the subject’s NSSLE on the front was almost straight, and the SSLE at the back was bent by 48° (additional information can be found in Supplementary Table S3, *t*_*3*_), and the struck-side elbow was raised and bent. If only the influence of the impact posture on the injury risk is considered, the NSSLE at the back may cause the body to rotate and result in more severe injury (Tang et al., 2020a). Pedestrians with a flexed knee in the pre-crash phase exhibit a lower injury risk (Li et al., 2015). In addition, an impact on the elbow influences head rotation, and the head undergoes rotational acceleration toward the vehicle (Paas et al., 2012). The pedestrian’s backward velocity ( $Vel_{y}$ ) affects the impact location with the vehicle and further affects the risk of injury (Elliott et al., 2012). The vertical acceleration ( $Acc_{y}$ ) of the pedestrian affects the load distribution on the lower limbs as well as the injury risk. In the FA case (Figure 3; Table 1), the pedestrian exhibited a running posture and had a higher forward velocity and vertical acceleration than that in the normal gait. The pedestrian head may directly collide with the ground over the vehicle front due to the high forward velocity, resulting in increased injury risk to the head. In such case, knee flexion in a running posture may reduce the injury risk of the leg (Li et al., 2015). In the case of OS avoidance (Figure 4; Table 1), the pedestrian faced the approaching vehicle and stepped backward; the impact posture and direction were thus different from those in the other cases. Since the pedestrian is usually facing with the vehicle in OS cases, the impact directions between the human body (head, chest, lower limbs et.) and the vehicle are unlike the side-impact under the normal gait, which causes the injury risks are different.

The joint angles, velocities, and accelerations of the pedestrians were accurately captured by the motion capture system. The peak value of the EMG signal corresponded to the peak value of acceleration in the process of avoidance behavior and was significantly higher than the EMG signal captured during a normal gait (Figures 2D, 3D, 4D; Supplementary Figure S4D). However, the influence of kinetic characteristics on pedestrian injury risk remains unclear. Pedestrian avoidance behavior is complex in terms of kinetic and kinematic features, which causes the impact conditions to be highly diverse and uncertain and also influences the pedestrian injury risk in vehicle collisions.

In addition, previous studies have not focused on the process of pedestrian avoidance behaviors (Soni et al., 2013a). When collisions occur during the process of pedestrian avoidance, the pedestrian’s posture, kinematics, and muscle activity will affect the risk of injury. Therefore, the influence of pedestrian avoidance behaviors on injury risk cannot be ignored. It is important to highlight the effect of pedestrian posture on the level of injury suffered from potential collisions. The data for pedestrian avoidance behaviors provided in this study can be used to analyze pedestrian injury mechanisms with a high-precision human numerical model (Golman et al., 2014). In addition, it can elucidate pedestrian injury characteristics in real-world accidents and provide data for predicting pedestrian trajectories and injury risks.

### 4.3 Application in HBM Development and Integrated Active and Passive Safety Systems

The presented kinematics and EMG data describe the pedestrian avoidance process completely. These data can serve as a reference for the development of more advanced biofidelic human models to predict pedestrian injury risk. For example, researchers can define the activation state of pedestrian muscles to develop an active human body model and analyze the influence of the muscle response on impact injuries.

In addition, the results of this study can facilitate the development of integrated active and passive safety systems from two aspects: collision risk assessment and potential injury prediction. Existing research has indicated that the development of vehicle active safety systems can improve the effectiveness of passive safety systems (Habibovic and Davidsson, 2012). To predict the potential injury risk of pedestrians, this study analyzed the pedestrian avoidance behaviors in detail and extracted three representative pedestrian pre-crash postures via normalization. As a database, these results can be used to input the collision condition into a numerical model to predict pedestrian injury risk and establish an injury risk prediction system. The pedestrian prediction system of a vehicle can currently only predict pedestrian behavior under normal walking states (Rasouli et al., 2018). This study describes the kinematic features of pedestrians, including the changes in velocity and acceleration and differences in posture relative to a normal gait that are essential information for quantitating pedestrian avoidance abilities and judging changes in pedestrian intentions.

### 4.4 Limitations

It should be noted that this study has several limitations. First, the scope was limited to the given representative traffic scenarios, which cannot represent all vehicle–pedestrian crash scenarios. Second, this study only focused on the behavioral characteristics of men aged 18–30 years; the behavioral characteristics of women, the elderly, and children need to be investigated in a future study. Third, although the current sample size could quantify pedestrian avoidance behaviors, a larger experimental sample size is necessary to predict the collision risk more accurately. Moreover, a follow-up study will focus on elucidating the relationship between pedestrian avoidance behaviors and the corresponding injury risks and severities. Furthermore, additional factors that influence pedestrian avoidance behaviors, such as age, gender, and stature, will be considered. A larger databank that includes a broad range of pedestrian active behavior characteristics in dangerous scenarios will be generated to predict the risk of collision more accurately.

## 5 Conclusion

This study identified pedestrian active avoidance behaviors and interaction processes with a vehicle in near-real traffic conflict scenarios using immersive VR technology. The time histories of kinetic and kinematic features of the pedestrian, such as velocity, acceleration, joint angles, EMG, and the relative location with the vehicle, were extracted to quantify and characterize the avoidance behaviors (all of the experimental data are available on an open-source platform). Pedestrian kinetics and kinematics at the instant of occurrence of collision were strongly influenced by the active avoidance behavior; for example, the pedestrian collision postures in the backward avoidance and forward avoidance behaviors were clearly and significantly different than that in the normal gait. In addition to the influence of individual physical conditions, sufficient time and a safe distance are also necessary for the pedestrian to perform a complete and effective avoidance motion. The minimum reaction time of pedestrians who successfully avoided collision was 1.6 s for BA behavior and 1.8 s for FA behavior. When investigating the effect of avoidance behaviors on injury risk and severity, the experimental data from this study can serve as a valuable reference for developing an FE/MB human model and simulating the pedestrian injury risk during collisions.

## Data Availability

The datasets presented in this study can be found in online repositories. The names of the repository/repositories and accession number(s) can be found below: https://github.com/QuanLI-21/Pedestrian-avoidance-behavior-dataset-PABD.
