# Supplementary material for: Kinetic and Kinematic Features of Pedestrian Avoidance Behavior in Motor Vehicle Conflicts
Source: Front Bioeng Biotechnol. 2021 Nov 25;9:783003. doi: 10.3389/fbioe.2021.783003 (PMC8655905; doi:10.3389/fbioe.2021.783003)
Supplement: Supplementary file 1 [file Table1.docx]

Supplementary Materials

Kinetic and kinematic features of pedestrian avoidance behavior in motor vehicle conflicts

Quan Li, Shi Shang, Xizhe Pei, Qingfan Wang, Qing Zhou, Bingbing Nie *

State Key Lab of Automotive Safety and Energy, School of Vehicle and Mobility, Tsinghua University, Beijing, China

*** Corresponding author:**

Bingbing Nie

E-mail address: nbb@tsinghua.edu.cn


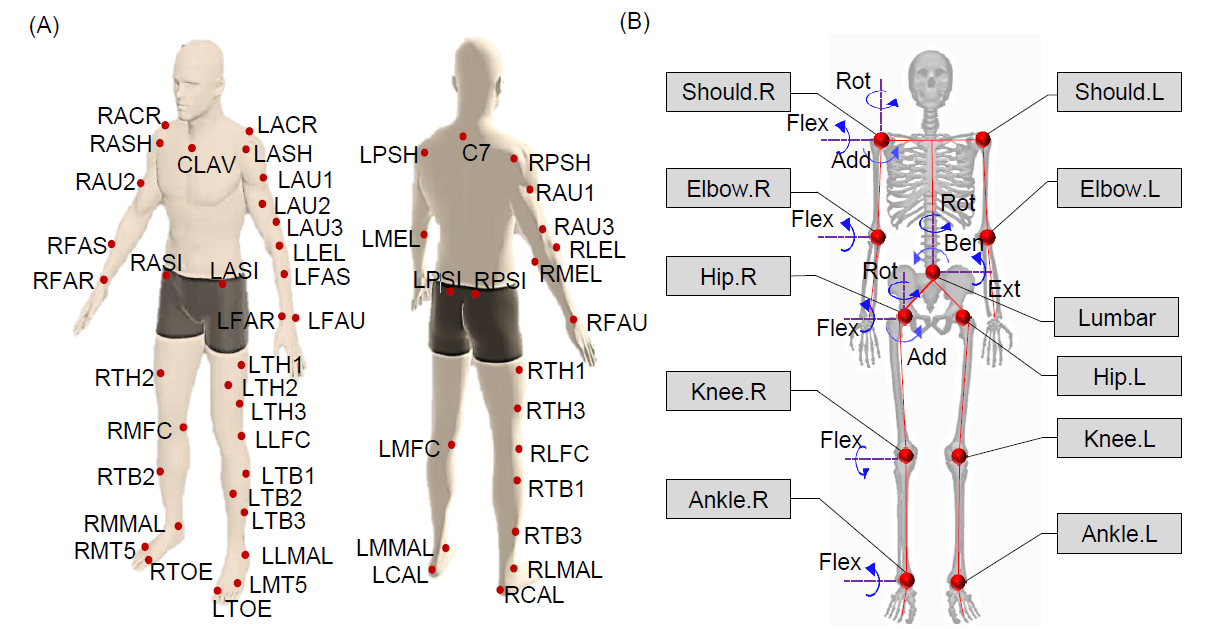


Figure S1. Definitions of marker locations and joint angles; (A) 54 marker locations, (B) Joints and rotation angles


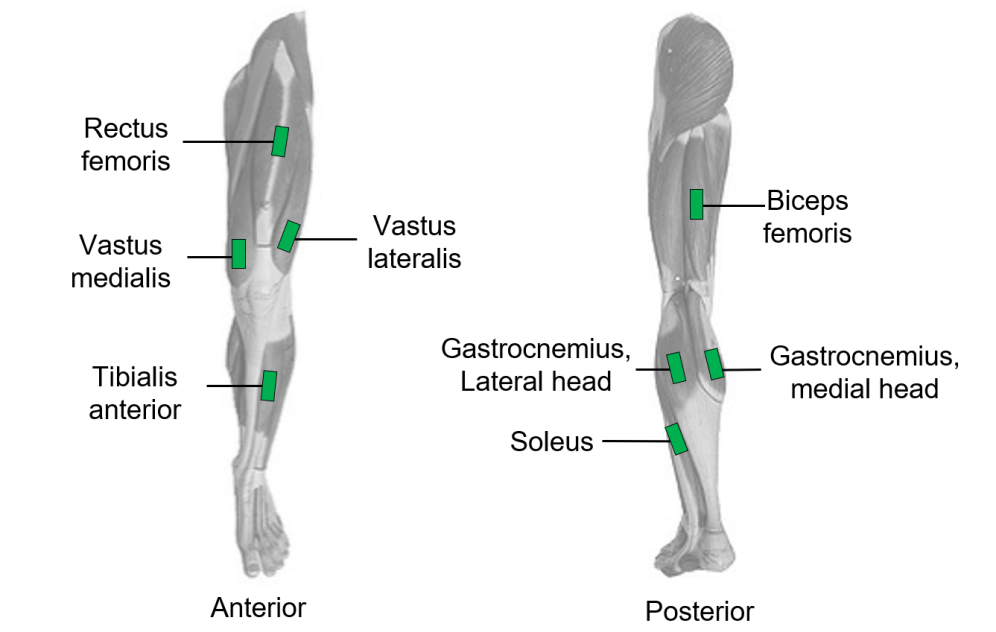


Figure S2. Locations of eight electrical signal sensors and measured muscles


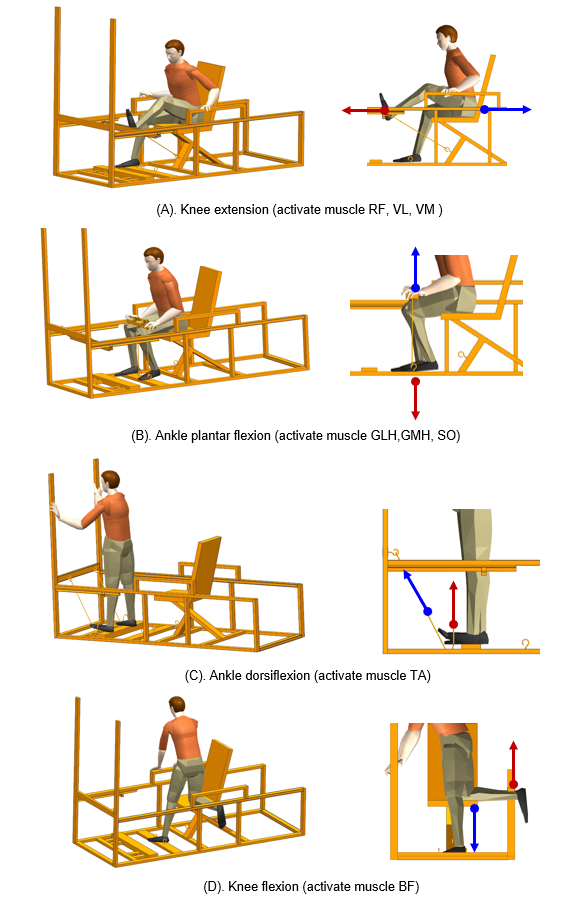


Figure S3. Maximum voluntary contraction (MVC) test methods


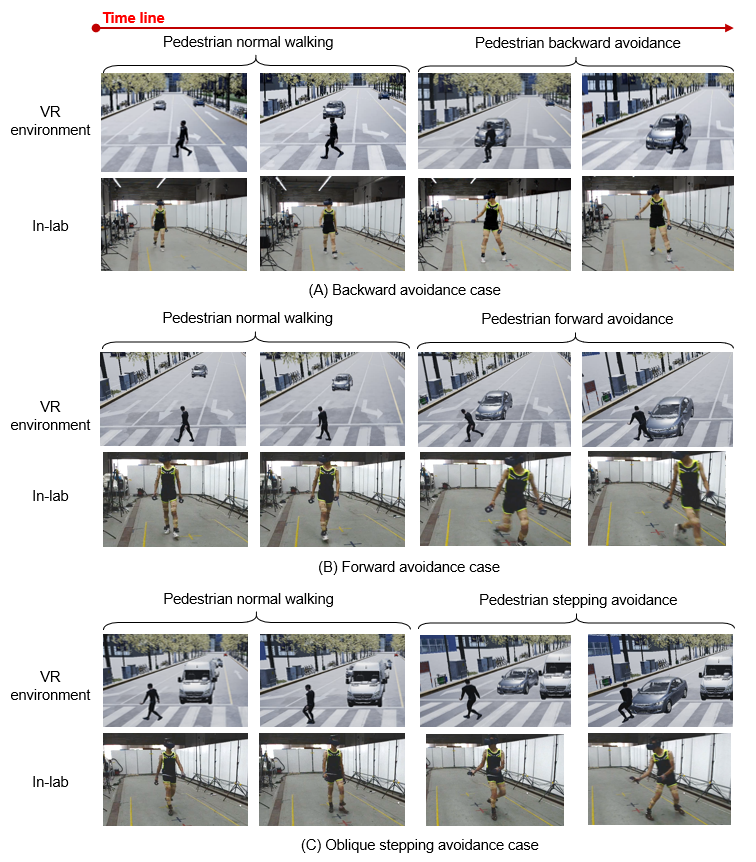


Figure S4. Pedestrian avoidance behaviors in case studies in the VR environment and in the laboratory


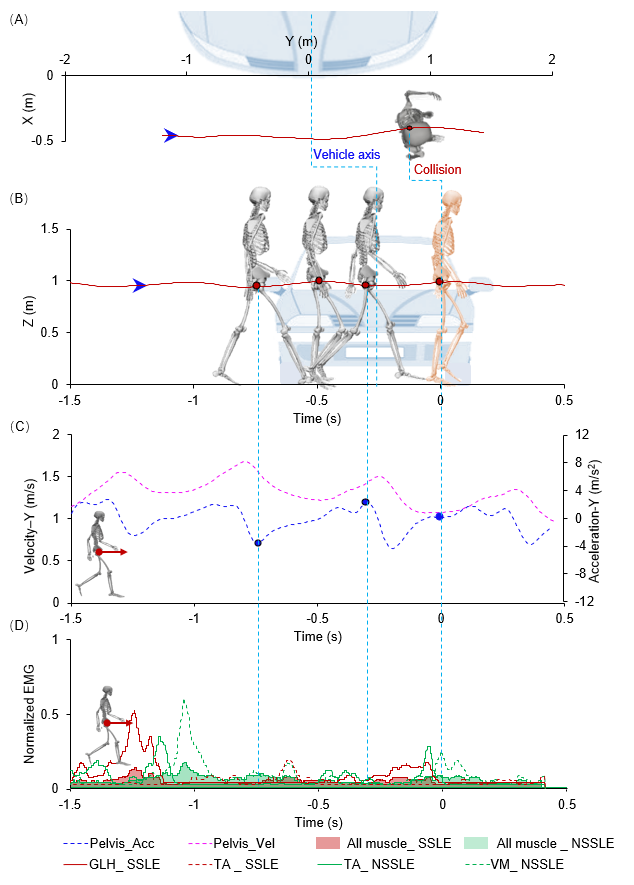


Figure S5. Pedestrian normal walking behavior (no avoidance reaction, NAR). (A) Top view of relative location of pedestrian and vehicle. (B) Side view of relative location of pedestrian and vehicle. (C) Pedestrian’s acceleration and velocity during avoidance. (D) Muscle activation level of pedestrian during avoidance.Time = 0 represents the collision time. In the above figures, the location of the vehicle at the collision time is shown. Negative times represent the time before the collision.

Table S1. Information for the 19 study participants

| Subj_No. | Gender | Age | High  (cm) | Weight  (kg) | BMI | Subj_No. | Gender | Age | High  (cm) | Weight  (kg) | BMI |
| --- | --- | --- | --- | --- | --- | --- | --- | --- | --- | --- | --- |
| 001 | Male | 22 | 171 | 70 | 23.9 | 016 | Male | 22 | 178 | 68 | 21.5 |
| 002 | Male | 24 | 170 | 75 | 26 | 017 | Male | 23 | 180 | 75 | 23.1 |
| 003 | Male | 22 | 174 | 70 | 23.1 | 018 | Male | 26 | 175 | 70 | 22.9 |
| 004 | Male | 22 | 170 | 55 | 19 | 019 | Male | 21 | 178 | 75 | 23.7 |
| 005 | Male | 21 | 178 | 65 | 20.5 | 021 | Male | 23 | 173 | 74 | 24.7 |
| 007 | Male | 21 | 172 | 60 | 20.3 | 022 | Male | 23 | 170 | 58 | 20.1 |
| 009 | Male | 24 | 174 | 55 | 18.2 | 023 | Male | 24 | 172 | 70 | 23.7 |
| 010 | Male | 18 | 173 | 55 | 18.4 | 024 | Male | 23 | 174 | 62 | 20.5 |
| 013 | Male | 21 | 169 | 57 | 20 | Average | / | 22.2 | 173.9 | 65.2 | 21.6 |
| 014 | Male | 20 | 183 | 65 | 19.4 | S.D. | / | ±1.8 | ±3.9 | ±7.3 | ±2.3 |
| 015 | Male | 21 | 170 | 60 | 20.8 |  |  |  |  |  |  |

Note: Subjects 006, 008, 011, 012, and 020 did not complete the experiment.

Table S2. Experiment results

| Subj_No. | Traffic scene | Noticed the “bullet vehicle” | Avoidance behavior | Collision | Traffic scene | Noticed the “bullet vehicle” | Avoidance behavior | Collision |
| --- | --- | --- | --- | --- | --- | --- | --- | --- |
| 001 | TSA | Yes | BA | No | / | / | / | / |
| 002 | TSA | No | No | Yes | / | / | / | / |
| 003 | TSA | Yes | BA | No | / | / | / | / |
| 004 | TSA | Yes | BA | No | / | / | / | / |
| 005 | TSA | Yes | BA | No | TSB | Yes | OS | Yes |
| 007 | TSA | Yes | FA | No | TSB | Yes | BA | No |
| 009 | TSA | No | No | Yes | TSB | Yes | BA | No |
| 010 | TSA | No | No | Yes | TSB | Yes | BA | No |
| 013 | TSA | Yes | BA | No | TSB | Yes | BA | No |
| 014 | TSA | No | No | No | TSB | Yes | FA | Yes |
| 015 | TSA | Yes | BA | No | TSB | No | No | Yes |
| 016 | TSA | Yes | OS | Yes | TSB | Yes | FA | No |
| 017 | TSA | No | No | Yes | TSB | Yes | FA | No |
| 018 | TSA | No | No | Yes | TSB | No | No | Yes |
| 019 | TSA | No | No | Yes | TSB | Yes | BA | Yes |
| 021 | TSA | Yes | FA | No | TSB | Yes | FA | No |
| 022 | TSA | No | No | Yes | TSB | Yes | BA | Yes |
| 023 | TSA | No | No | Yes | TSB | Yes | FA | Yes |
| 024 | TSA | No | No | Yes | TSB | Yes | BA | No |

Note: Subjects 001, 002, 003, and 004 only participated in TSA.

Table S3. Joint angles of pedestrian avoidance postures in backward avoidance (Fig. 2) (unit: degrees; *t_3_* is the collision time)

| Joint | | *t_1_* | *t_2_* | *t_3_* | *t_4_* | Joint | | *t_1_* | *t_2_* | *t_3_* | *t_4_* |
| --- | --- | --- | --- | --- | --- | --- | --- | --- | --- | --- | --- |
| Struck-side lower extremity (SSLE) | Should-Flex | -5.7 | 1.0 | 9.1 | 13.9 | Non-struck-side lower extremity (NSSLE) | Should-Flex | 3.6 | 6.8 | -8.9 | -6.9 |
|  | Should-Add | -19.0 | -24.9 | -38.1 | -42.5 |  | Should-Add | -28.4 | -29.8 | -16.4 | -20.9 |
|  | Should-Rot | 19.2 | -7.4 | -6.1 | -3.5 |  | Should-Rot | -4.9 | 0.8 | -15.5 | -14.5 |
|  | Elbow-Flex | 21.4 | 72.4 | 75.8 | 77.8 |  | Elbow-Flex | 48.4 | 52.5 | 55.6 | 56.0 |
|  | Hip-Flex | 24.5 | 23.9 | 14.6 | 17.0 |  | Hip-Flex | -5.4 | 24.1 | 27.3 | 29.6 |
|  | Hip-Add | -6.0 | -1.3 | -5.2 | -4.8 |  | Hip-Add | 1.0 | -23.0 | -18.9 | -14.9 |
|  | Hip-Rot | -4.0 | 10.4 | 2.3 | -3.7 |  | Hip-Rot | -10.2 | 10.0 | -1.2 | -9.0 |
|  | Knee-Flex | 6.4 | 12.9 | 48 | 35.5 |  | Knee-Flex | 27.3 | 33.5 | 0.6 | 11.5 |
|  | Ankle-Flex | -26.1 | -23.4 | 6.7 | 13.6 |  | Ankle-Flex | 21.5 | -7.6 | -38.2 | -24.9 |
| Lumbar-Ext | | -10.5 | -16.2 | -9.8 | -11.7 | Pelvis_x | | 2.2 | -5.6 | -0.5 | 4.3 |
| Lumbar-ben | | -6.6 | -3.8 | -2.8 | -5.4 | Pelvis_y | | 2.1 | 0.8 | 0.7 | -6.6 |
| Lumbar-Rot | | -24.9 | -18.3 | -6.1 | -8.2 | Pelvis_z | | -1.5 | -25.1 | -23.3 | -28.2 |

Table S4. Joint angles of pedestrian avoidance postures in forward avoidance (Fig. 3) (unit: degrees; *t_3_* is the collision time)

| Joints | | *t_1_* | *t_2_* | *t_3_* | Joins | | *t_1_* | *t_2_* | *t_3_* |
| --- | --- | --- | --- | --- | --- | --- | --- | --- | --- |
| Struck side lower extremity (SSLE) | Should-Flex | 16.1 | 1.3 | -15.2 | Nonstruck side lower extremity (NSSLE) | Should-Flex | -19.2 | 17.4 | 20.6 |
|  | Should-Add | -17.3 | -18.3 | -16.3 |  | Should-Add | -15.9 | -32.8 | -22.6 |
|  | Should-Rot | 17.7 | -10.6 | -15.8 |  | Should-Rot | -14.3 | -0.6 | -0.5 |
|  | Elbow-Flex | 33.4 | 45.7 | 52.2 |  | Elbow-Flex | 41.1 | 66.9 | 71.2 |
|  | Hip-Flex | -16.6 | 42.4 | 46.2 |  | Hip-Flex | 37.1 | 15.6 | -5.9 |
|  | Hip-Add | -14.1 | 6.1 | 2.8 |  | Hip-Add | -25.6 | -3.6 | 4.9 |
|  | Hip-Rot | 1.8 | 4.0 | -7.2 |  | Hip-Rot | -18.5 | -12.7 | -15.7 |
|  | Knee-Flex | 3.4 | 85.4 | 30.0 |  | Knee-Flex | 20.0 | 50.2 | 34.4 |
|  | Ankle-Flex | 9.2 | 5.9 | -3.0 |  | Ankle-Flex | -6.6 | 27.6 | -19.5 |
| Lumbar-Ext | | -7.4 | -11.4 | -4.4 | Pelvis_x | | -0.4 | -2.3 | 1.5 |
| Lumbar-ben | | -6.2 | 2.0 | 5.6 | Pelvis_y | | -5.1 | 7.0 | -6.5 |
| Lumbar-Rot | | 6.1 | -11.5 | -23.2 | Pelvis_z | | -11.1 | -14.5 | -10.2 |

Table S5. Joint angles of pedestrian avoidance postures in oblique stepping avoidance (Fig. 4) (unit: degrees; *t_2_* is the collision time)

| Joint | | *t_1_* | *t_2_* | Joint | | *t_1_* | *t_2_* |
| --- | --- | --- | --- | --- | --- | --- | --- |
| Struck-side lower extremity (SSLE) | Should-Flex | -8.9 | 51.0 | Non-struck-side lower extremity (NSSLE) | Should-Flex | 27.8 | 49.1 |
|  | Should-Add | -14.7 | -34.3 |  | Should-Add | -16.9 | -8.7 |
|  | Should-Rot | -28.6 | -26.9 |  | Should-Rot | 10.1 | 47.4 |
|  | Elbow-Flex | 31.8 | 33.5 |  | Elbow-Flex | 49.9 | 77.4 |
|  | Hip-Flex | 13.7 | 14.9 |  | Hip-Flex | -28.3 | -1.4 |
|  | Hip-Add | -0.6 | -26.1 |  | Hip-Add | -0.3 | -4.0 |
|  | Hip-Rot | -19.7 | 2.6 |  | Hip-Rot | -29.4 | -23.3 |
|  | Knee-Flex | 5.7 | 26.0 |  | Knee-Flex | 16.6 | 34.2 |
|  | Ankle-Flex | -25.6 | -14.1 |  | Ankle-Flex | 3.6 | 16.0 |
| Lumbar-Ext | | -21.0 | -32.8 | Pelvis_x | | 0.0 | 0.6 |
| Lumbar-ben | | -2.4 | 23.9 | Pelvis_y | | 11.4 | 6.9 |
| Lumbar-Rot | | -26.4 | -4.1 | Pelvis_z | | 0.0 | -28.1 |

Table S6. Joint angles of pedestrian emergency posture (Fig. 5) (unit: degrees)

| Joint | | *t_1_* | *t_2_* | *t_3_* | Joint | | *t_1_* | *t_2_* | *t_3_* |
| --- | --- | --- | --- | --- | --- | --- | --- | --- | --- |
| Struck-side lower extremity (SSLE) | Should-Flex | 13.3 | 9 | 24.3 | Non-struck-side lower extremity (NSSLE) | Should-Flex | 2.1 | 5.3 | -7 |
|  | Should-Add | -17.1 | -15.7 | -20 |  | Should-Add | -15.5 | -16.3 | -17 |
|  | Should-Rot | 3.1 | -1.8 | 5.3 |  | Should-Rot | -4.4 | -4.9 | -11.8 |
|  | Elbow-Flex | 64.1 | 61.9 | 56 |  | Elbow-Flex | 38.6 | 49.7 | 61.5 |
|  | Hip-Flex | -8.9 | 3.8 | -2.3 |  | Hip-Flex | -4.9 | 14.9 | 34 |
|  | Hip-Add | -2.8 | -9.6 | -4 |  | Hip-Add | -10.5 | -17.8 | -25.6 |
|  | Hip-Rot | -6.7 | 11.5 | -22.1 |  | Hip-Rot | -14.2 | -15.1 | -12.6 |
|  | Knee-Flex | 36.8 | 59.6 | 29.1 |  | Knee-Flex | 19.7 | 27.8 | 50.7 |
|  | Ankle-Flex | 11.9 | 13.4 | 12.9 |  | Ankle-Flex | -2.3 | -2.2 | 12.3 |
| Lumbar-Ext | | -42 | -26.4 | -21.5 | Pelvis_x | | -11.2 | 7.9 | -9 |
| Lumbar-ben | | -0.5 | -7.6 | 10.1 | Pelvis_y | | 20.2 | -6.7 | 0.9 |
| Lumbar-Rot | | -0.6 | 2.5 | -0.2 | Pelvis_z | | -14 | -39 | -14.8 |
